# Network Meta-Analysis of Exercise Interventions on Working Memory Capacity in College Students: A Comparative Study Based on Intervention Types

**DOI:** 10.3390/bs16050711

**Published:** 2026-05-06

**Authors:** Shenning Zhou, Mengyao Feng, Yueyan Li, Xiangqin Song

**Affiliations:** College of Physical Education and Sports, Beijing Normal University, Beijing 100875, China; 202521070008@mail.bnu.edu.cn (S.Z.); mayo1121@mail.bnu.edu.cn (M.F.); 202521070027@mail.bnu.edu.cn (Y.L.)

**Keywords:** network meta-analysis, exercise interventions, working memory, college students, HIIT, cognitive engagement

## Abstract

**Background**: Working memory is essential for college students’ academic success, yet modern sedentary and digitally distracted lifestyles threaten cognitive health. This network meta-analysis compared the effectiveness of six exercise modalities on working memory in this population. **Methods**: Following PRISMA-NMA guidelines (PROSPERO: CRD420261331066), we systematically searched PubMed, Web of Science, Embase, Cochrane Library, SPORTDiscus, and CNKI for randomized controlled trials up to 13 February 2026. The primary outcome was reaction time on working memory tasks. We used frequentist random-effects network meta-analysis in Stata 18.0. **Results**: Thirty-five trials (2314 participants) were included. Compared to controls, significant benefits were found for aerobic exercise (SMD = −0.63, 95% CI: −0.94 to −0.32), mixed-modal training (SMD = −0.59, 95% CI: −0.92 to −0.26), HIIT (SMD = −0.56, 95% CI: −0.96 to −0.16), and resistance training (SMD = −0.48, 95% CI: −0.82 to −0.14). Ball sports and cognitive training showed no significant effects. HIIT and mixed-modal training ranked highest (SUCRA 80.3% and 76.0%). Chronic interventions (≥4 weeks) yielded larger effects than acute protocols. Direct comparisons among effective modalities revealed no significant differences. **Conclusions**: Aerobic-dominant exercise interventions were associated with moderate working memory improvements in college students, though the optimal type remains uncertain pending further comparative trials.

## 1. Introduction

College students are at a critical stage of cognitive development marked by ongoing prefrontal cortex maturation essential for academic achievement and professional readiness. Within this context, working memory (WM)—comprising specialized storage systems and a central executive per Baddeley’s multicomponent model—is a cornerstone for information encoding, logical reasoning, and academic performance. Extensive evidence confirms that WM capacity robustly predicts fluid intelligence and scholastic outcomes, including GPA and standardized test scores (Alloway & Alloway, 2010; Baddeley, 2003; Kyllonen & Christal, 1990).

However, this critical cognitive function faces increasing challenges in contemporary higher education environments. The confluence of elevated sedentary behavior and pervasive digital multitasking among college students has been linked to attenuated prefrontal activation and diminished WM updating efficiency (Gogniat et al., 2025; Loh & Kanai, 2016). Given these threats, identifying safe, scalable, and effective interventions to bolster cognitive resilience in this population is an urgent priority.

Physical activity interventions represent a promising non-pharmacological strategy for cognitive enhancement, known to improve cerebral perfusion and upregulate neurotrophic factors (Colcombe & Kramer, 2003). Despite this potential, research in college cohorts remains fragmented. On one hand, existing evidence predominantly focuses on single exercise modalities (e.g., aerobic exercise or resistance training alone), lacking cross-sectional comparisons among different intervention types. For example, can time-efficient interventions represented by high-intensity interval training (HIIT) yield cognitive benefits comparable to traditional moderate-intensity continuous training? Do open-skill activities with high cognitive engagement (e.g., ball sports, dance) better activate executive function networks compared to closed-skill activities (e.g., running)? These critical questions regarding comparative effectiveness remain unresolved. On the other hand, traditional pairwise meta-analyses can only synthesize direct comparison evidence, failing to integrate the mixed network relationships among multiple intervention types and unable to rank the effectiveness of interventions, thereby limiting the translation of research findings into precise exercise prescription guidelines (Salanti et al., 2014).

Furthermore, different types of exercise may exert distinct influences on the subcomponents of working memory. Some theories suggest that aerobic exercise primarily enhances neural efficiency by improving cerebrovascular function, thereby promoting information processing speed; whereas resistance training or cognitively engaging activities may expand storage capacity by strengthening executive control (Kleinloog et al., 2019). However, existing research findings are inconsistent, and a systematic integrative analysis is lacking. Clarifying the differential effects of various intervention types on the “storage” and “processing” subcomponents of working memory holds significant theoretical value for developing targeted cognitive enhancement strategies.

In light of this, the present study employs a network meta-analysis approach to systematically compare the relative effectiveness of different physical activity interventions (e.g., aerobic exercise, resistance training, HIIT, ball sports, etc.) on working memory in college students. A network meta-analysis is particularly suited to this question because it allows for the simultaneous comparison of multiple interventions using both direct and indirect evidence, thereby generating a coherent ranking of treatment options even when head-to-head trials are sparse—a common scenario in exercise–cognition research. By integrating direct and indirect evidence, this study aims to address core questions that cannot be answered by single-modality exercise studies: Which type of exercise is most effective in improving working memory in college students? Are there significant differences among different intervention types? The findings will provide a solid evidence-based foundation for the reform of university physical education curricula and the development of personalized cognitive health promotion programs for college students. Furthermore, by distinguishing between acute and chronic intervention effects, this study aims to clarify the temporal specificity of exercise-induced cognitive benefits.

## 2. Materials and Methods

### 2.1. Search Strategy, Inclusion, and Screening

This review was conducted in strict accordance with the Preferred Reporting Items for Systematic Reviews and Meta-Analyses for Network Meta-Analyses (PRISMA-NMA) guidelines (Hutton et al., 2015) and was prospectively registered with PROSPERO (CRD420261331066). The search strategy was developed based on a combination of Medical Subject Headings (MeSH, 2025) and free-text terms, structured around three key concepts: (1) college students/young adults; (2) exercise/physical activity interventions; and (3) working memory/cognitive function. Logical operators (AND, OR) were used to appropriately combine these components. The full electronic search strategy for PubMed was as follows: (“Exercise”[Mesh] OR “Motor Activity”[Mesh] OR “Sports”[Mesh] OR “Physical Education and Training”[Mesh] OR “Physical Training”[tiab] OR “Sports”[tiab] OR “Exercise”[tiab] OR “Aerobic Exercise”[tiab] OR “Open Skill”[tiab] OR “Closed Skill”[tiab] OR “Team Sport”[tiab] OR “Individual Sport”[tiab]) AND (“Executive Function”[Mesh] OR “Cognition”[Mesh] OR “Executive Function”[tiab] OR “Executive Control”[tiab] OR “Inhibitory Control”[tiab] OR “Cognitive Flexibility”[tiab] OR “Working Memory”[tiab] OR “Cognitive Performance”[tiab]) AND (“Students”[Mesh] OR “Young Adult”[Mesh] OR “Adult”[Mesh] OR “College Student”[tiab] OR “University Student”[tiab] OR “Undergraduate”[tiab] OR “Young Adult”[tiab]). This strategy was adapted for each database (Web of Science, Embase, Cochrane Library, SPORTDiscus, and CNKI) according to its specific syntax and controlled vocabulary. 

A systematic search was conducted in health-related, biomedical, and psychological databases (PubMed, Web of Science, Embase, Cochrane Library, SPORTDiscus, and CNKI) up to 13 February 2026. Additionally, the reference lists of relevant original studies and reviews were screened, but no additional studies were identified.

**Figure 1:**
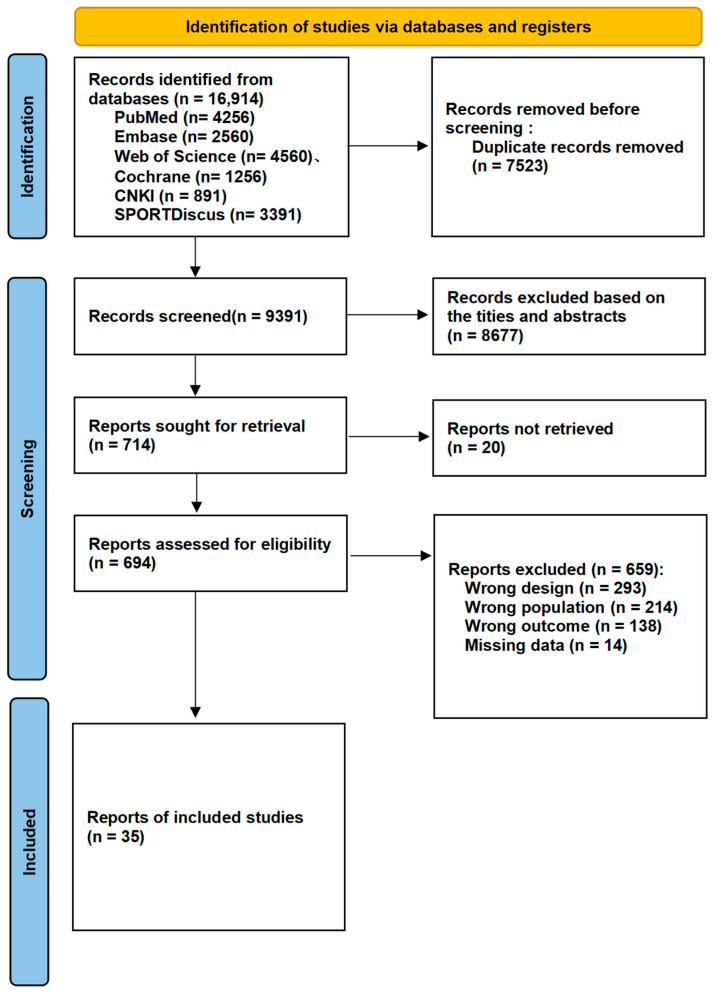
Literature screening process for the effects of different exercise modalities on working memory in college students.

After the removal of duplicate records, the researchers conducted a stepwise screening of the remaining literature using the following three-stage criteria: (1) title, (2) abstract, and (3) full text (see Figure 1). The final decisions regarding inclusion or exclusion were made jointly by two independent reviewers (ZSN, FMY), with any disagreements resolved by a third reviewer (SXQ). The study employed the PICOS framework [Population, Intervention, Comparison, Outcomes, and Study Design] as follows: (1) Population (P)—full-time college students (including undergraduate and graduate students), aged 18 years and above, with no restrictions on gender, nationality, or major. Special populations with severe mental illness, neurological disorders, or physical disabilities that could affect cognitive function testing or exercise performance were excluded. (2) Intervention (I)—various structured physical activity interventions, including but not limited to aerobic exercise, resistance training, high-intensity interval training (HIIT), and cognitively engaging activities (e.g., ball sports, dance, martial arts). Parameters of the interventions (frequency, intensity, duration) were not restricted, but clear descriptions of the exercise prescription were required. (3) Comparison (C)—studies that included no-intervention (blank control), usual lifestyle (waitlist), passive control (e.g., health education, reading), or active control (e.g., stretching, low-intensity activities) conditions. Given the network meta-analysis design of this study, multi-arm studies with significant differences in exercise type or intensity between intervention groups (e.g., HIIT group versus moderate-intensity aerobic group) were also eligible, with each considered as a separate node for comparison. (4) Outcomes (O)—at least one objective, standardized, quantifiable measure of working memory. Primary outcomes needed to be assessed using classic neuropsychological tests, such as the digit span test (measuring working memory storage capacity), the N-back task (measuring executive function), or the Corsi block-tapping task, with means and standard deviations reported at baseline and post-intervention. (5) Study Design (S)—randomized controlled trials (RCTs). Studies were required to report sufficient statistical data (e.g., means, standard deviations, sample sizes) to calculate effect sizes such as the standardized mean difference (SMD). Non-randomized controlled trials, reviews, case reports, and conference abstracts with insufficient data for extraction were excluded.

### 2.2. Quality Assessment

The methodological quality (including risk of bias) of the included experimental studies was assessed using the PEDro scale (Physiotherapy Evidence Database) (Maher et al., 2003). The PEDro scale was selected because it is a widely adopted and validated tool for assessing methodological quality in exercise intervention trials, with established reliability for rating randomized controlled designs. This scale comprises 11 dichotomous (yes/no) items, with items 2–9 assessing randomization/internal validity and items 10–11 assessing the presence of statistically replicable results. Item 1 pertains to external validity but is not used in the calculation of the PEDro score. Studies were independently evaluated by two authors (ZSN, FMY), with any disagreements resolved by a third author (SXQ). PEDro scores range from 0 to 10, with one point awarded for each satisfied criterion. Quality classification was as follows: <4 = poor, 4–5 = fair, 6–8 = good, and 9–10 = excellent. A score of 6 or above indicates high study quality (Maher et al., 2003).

### 2.3. Data Extraction

Relevant data were independently extracted by two researchers (ZSN, FMY) and entered into a predesigned Excel spreadsheet. The extracted information primarily included basic study characteristics (authors, year), the number of participants in each group, and the means and standard deviations of pre-test and post-test scores on working memory tasks. For tasks involving multiple cognitive load conditions, the condition with higher cognitive demand was prioritized (e.g., selecting 3-back over 2-back in the N-back task; selecting complex span tasks over simple span tasks). This is because high-demand conditions are less susceptible to ceiling effects and are more sensitive to detecting intervention-induced changes in young adult populations. The classification of working memory tasks was based on Baddeley’s multicomponent model of working memory (Baddeley, 2003) and was verified by a neuropsychology expert.

Selection of Outcome Measures: The target population comprised healthy college students, a demographic known to perform at high levels on standard working memory tasks. Extensive evidence indicates that accuracy measures (e.g., percentage correct in N-back or digit span tasks) frequently approach ceiling in this group, exhibiting restricted variance and thus limited sensitivity to intervention-induced changes (Jaeggi et al., 2010; Wiemers et al., 2019). In our preliminary screening, mean accuracy rates were uniformly high (typically exceeding 85–90%) with small standard deviations, confirming the presence of ceiling effects. Consequently, when a study reported both accuracy and reaction time, reaction time was prioritized as the primary outcome to maximize statistical sensitivity. Accuracy data were not consistently extracted across all studies due to variability in reporting formats; therefore, no formal sensitivity analysis using accuracy could be conducted. This limitation is explicitly addressed in the Discussion. If data were only presented in graphs, numerical values were extracted using WebPlotDigitizer version 4.5 (Drevon et al., 2017).

Interventions were classified into six categories based on their energy system activation and cognitive engagement: aerobic exercise, resistance training, high-intensity interval training (HIIT), ball sports (as cognitively engaging activities), cognitive training, and mixed-modal training. Control groups were categorized as passive control or active control. Disagreements during the data extraction process were resolved through discussion; if consensus could not be reached, a third researcher (SXQ) was consulted. All extracted data were cross-checked and then imported into Stata 18.0 software for analysis. (A complete list of all included studies is provided in Appendix A.)

### 2.4. Statistical Analysis

Pre- and post-intervention means and standard deviations were used to calculate the standardized mean difference (SMD) with 95% confidence intervals. Bias-corrected Hedges’ g was used to adjust for small-sample bias (Hedges, 1981). If studies employed multiple cognitive tasks, data were combined into a single outcome.

Definition of Effect Size Direction: For reaction time measures, lower values indicate better performance (higher processing efficiency). To facilitate the interpretation of results, this study stipulated that a negative SMD (SMD < 0) indicates shorter reaction time in the intervention group compared to the control group, meaning the intervention effect is superior to the control; the larger the absolute value of the SMD, the greater the effect. Data analysis was conducted within a frequentist framework. Network meta-analysis models were constructed and fitted using a random-effects model with Stata 18.0 software, with the “inactive control group” as the reference comparator. The analysis included: (1) generating a network plot to illustrate the direct and indirect relationships among interventions; (2) generating a contribution plot to quantify the weight of direct comparisons in the mixed estimates; (3) assessing heterogeneity using the I^2^ statistic; and (4) testing network inconsistency using the node-splitting method. Forest plots were constructed to display the effect sizes, and the magnitude of effects was interpreted according to Cohen’s guidelines (Dias et al., 2010). The ranking of intervention effectiveness was based on the P-score ranking metric (Rücker & Schwarzer, 2015), with values closer to 1 indicating a more favorable intervention. Furthermore, the risk of publication bias was visually assessed by examining the asymmetry of comparison-adjusted funnel plots (Chaimani & Salanti, 2012).

### 2.5. Subgroup Analysis by Intervention Duration

Given the fundamental neurophysiological distinction between transient effects of acute exercise (e.g., immediate catecholamine release, augmented cerebral blood flow) and sustained adaptations induced by chronic training (e.g., upregulation of BDNF, synaptic plasticity), we conducted a pre-planned subgroup analysis stratified by intervention duration. Studies were categorized into two mutually exclusive subgroups: acute subgroup—intervention duration < 4 weeks (including single-session protocols; *n* = 19 studies); and chronic subgroup—intervention duration ≥ 4 weeks (*n* = 16 studies). For each subgroup, network connectivity was verified prior to analysis. The acute subgroup formed a fully connected network including six treatment nodes (aerobic, resistance, HIIT, cognitive, mixed, and passive control) with 19 studies. For the chronic subgroup, six studies were identified as containing only a single treatment arm after excluding acute protocols and were therefore removed to ensure network connectivity. The remaining 10 studies formed a connected network comprising six nodes (aerobic, resistance, HIIT, ball sports, mixed, and passive control). Although the chronic network had fewer studies and one different node (ball sports replacing cognitive training), both subnetworks were fully connected and suitable for indirect comparison inference within a consistency model. The reduced sample size in each subgroup, particularly the chronic subgroup, necessarily increases the uncertainty of estimates, as reflected in wider confidence intervals for some comparisons. This limitation is further addressed in the Discussion (Section 4.6). Within each subgroup, a separate frequentist network meta-analysis was performed using identical statistical specifications as the primary analysis (random-effects model, Hedges’ g as effect measure, passive control as reference). Results are presented in Section 3.4.

## 3. Results

### 3.1. Literature Screening and Inclusion Results

Following the PRISMA guidelines, we conducted literature screening and selection. A systematic search of PubMed, Web of Science, Embase, Cochrane Library, SPORTDiscus, and CNKI databases yielded a total of 16,914 initial records. After removing 7523 duplicate records, the titles and abstracts of the remaining 9391 articles were preliminarily screened, resulting in the exclusion of 8677 articles that did not meet the inclusion criteria. Subsequently, we attempted to retrieve the full texts of the remaining 714 articles, of which 20 could not be obtained. A detailed evaluation was performed on the 694 successfully retrieved full texts, leading to the exclusion of 659 articles that did not meet the criteria. The primary reasons for exclusion included: the study population not being college students, unclear descriptions of the intervention measures, absence of objective working memory outcome measures, non-randomized controlled trial study designs, and insufficient statistical data. Additionally, the reference lists of relevant original studies and reviews were screened, but no additional studies were identified. Ultimately, a total of 35 studies were included in this network meta-analysis. All included studies were randomized controlled trials evaluating the effects of exercise interventions on working memory in college students. The entire screening process was conducted by two independent researchers (ZSN, FMY), with disagreements resolved by a third researcher (SXQ). Figure 1 illustrates the detailed literature screening process.

### 3.2. Basic Characteristics of Included Studies

The 35 included studies involved 2314 participants from multiple countries. All participants were full-time college students with normal baseline cognitive function. Study quality was relatively high (PEDro scores: 5–10; mean ± SD: 7.0 ± 1.4). Interventions were classified into six categories: resistance training, aerobic exercise, HIIT, ball sports, cognitive training, and mixed-modal training. The intervention duration ranged from a single acute session to 16 weeks, with frequencies of 1–5 sessions per week and session durations of 10–60 min. Control groups primarily consisted of no-intervention or passive control conditions. Outcome assessments employed standardized neuropsychological tests (N-back, digit span, computation span).

### 3.3. Network Meta-Analysis Results

A frequentist network meta-analysis was conducted using the network package in Stata 18.0. Using the control group (treatment 6) as the common reference, the standardized mean difference (SMD) and 95% confidence interval were calculated for each intervention relative to the control group. The model assumed that the between-study effects followed a multivariate normal distribution with a compound symmetry covariance structure, and a consistency model was fitted using the mvmeta command.

#### 3.3.1. Network Geometry

**Figure 2:**
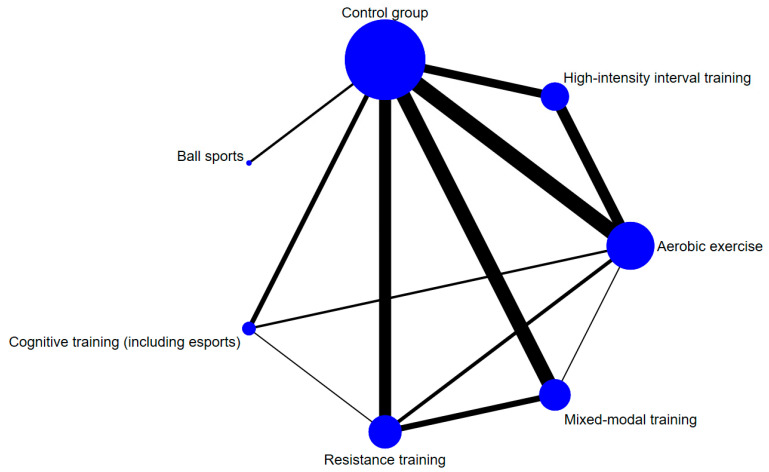
Network evidence of the effects of different exercise modalities on working memory in college students.

Figure 2 illustrates the direct comparison relationships between different types of exercise interventions (resistance training, aerobic exercise, high-intensity interval training, ball sports, cognitive training, and mixed-modal training) and the control group. The node size reflects the total sample size for each intervention. The control group had the largest node (*n* = 826), followed by aerobic exercise (*n* = 383), mixed-modal training (*n* = 382), and resistance training (*n* = 319), indicating that these interventions were most common in the included studies. The thickness of the lines represents the number of studies providing direct comparisons. The lines between aerobic exercise and the control group (13 studies), resistance training and the control group (10 studies), mixed-modal training and the control group (12 studies), and aerobic exercise and high-intensity interval training (9 studies) were thicker, suggesting that the most substantial direct evidence existed for these comparisons. In contrast, direct comparison data involving ball sports and cognitive training with other interventions were relatively sparse (e.g., only two studies for ball sports vs. control; two studies for cognitive training vs. aerobic exercise; one study for cognitive training vs. resistance training). The limited number of closed loops in the network plot indicates a relative scarcity of direct comparisons among the interventions themselves, with most studies focusing on comparisons between active interventions and the control group. This provides a crucial foundation for integrating indirect evidence into the network meta-analysis.

#### 3.3.2. Heterogeneity and Network Consistency Assessment

Overall heterogeneity was moderate (τ = 0.53, I^2^ ≈ 34%). The design-by-treatment interaction test detected no significant inconsistency (χ^2^ = 10.93, *p* = 0.535). Node-splitting analysis showed no significant differences between direct and indirect estimates for all comparisons (all *p* > 0.05). Local inconsistency assessment using loop-specific inconsistency factors identified significant inconsistency only in the resistance–aerobic–mixed-modal loop (A-B-G: IF = 1.11, 95% CI: 0.15 to 2.08, *p* = 0.024), likely due to limited direct evidence and definitional variability. Overall, the network consistency assumption remains acceptable.

#### 3.3.3. Comparison of the Effects of Exercise Interventions on Working Memory

The network meta-analysis, using the control group as the reference, showed that (Table 1) aerobic exercise (SMD = −0.63, 95% CI: −0.94 to −0.32; *p* < 0.001), high-intensity interval training (HIIT) (SMD = −0.56, 95% CI: −0.96 to −0.16; *p* = 0.007), and mixed-modal training (SMD = −0.59, 95% CI: −0.92 to −0.26; *p* < 0.001) were all significantly superior to the control group, with moderate effect sizes. Resistance training was also significantly superior to the control group (SMD = −0.48, 95% CI: −0.82 to −0.14; *p* = 0.006). Ball sports (SMD = −0.06, 95% CI: −0.88 to 0.76; *p* = 0.885) and cognitive training (SMD = −0.09, 95% CI: −0.61 to 0.43; *p* = 0.733) showed no statistically significant difference compared to the control group. Given that estimates for ball sports and cognitive training were informed by only two and four studies, respectively, these findings should be regarded as preliminary and interpreted with considerable caution.

**Figure 3:**
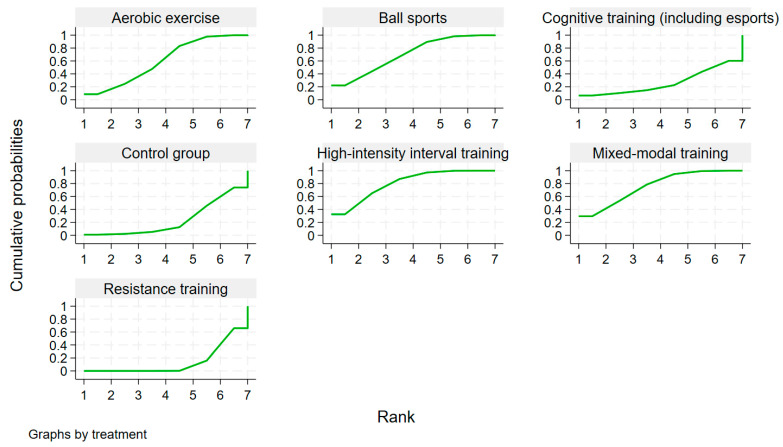
Ranking probability distribution of the effects of different exercise modalities on working memory in college students.

SUCRA rankings (Table 1 and Figure 3) indicated that HIIT ranked highest (SUCRA = 80.3%, probability best = 32.5%), followed closely by mixed-modal training (76.0%, 29.5%). Ball sports ranked third (70.1%), aerobic exercise fourth (60.3%), cognitive training fifth (26.2%), and resistance training lowest among active interventions (13.7%). It is important to note that SUCRA rankings reflect probabilistic ordering and should be interpreted in conjunction with pairwise effect estimates and confidence intervals. As detailed in the Discussion (Section 4.1), the apparent discrepancy between ranking positions and statistical significance underscores the exploratory nature of these rankings.

#### 3.3.4. League Table of Pairwise Comparisons Among All Interventions

The league table results of the network meta-analysis (Table 2) present the pairwise comparisons among all interventions. Using the control group as the reference, aerobic exercise (SMD = −0.63, 95% CI: −0.94 to −0.31), mixed-modal training (SMD = −0.59, 95% CI: −0.92 to −0.26), HIIT (SMD = −0.56, 95% CI: −0.96 to −0.16), and resistance training (SMD = −0.48, 95% CI: −0.82 to −0.14) all significantly reduced reaction time on working memory tasks, with moderate effect sizes (|SMD| = 0.48 to 0.63). In contrast, ball sports (SMD = −0.06, 95% CI: −0.88 to 0.76) and cognitive training (SMD = −0.09, 95% CI: −0.60 to 0.43) showed no statistically significant difference compared to the control group, suggesting that the efficacy of these two interventions in improving working memory processing efficiency in college students remains inconclusive.

Direct comparisons among the four effective interventions revealed no significant differences: aerobic exercise vs. mixed-modal training (SMD = −0.03, 95% CI: −0.47 to 0.40), aerobic exercise vs. HIIT (SMD = −0.07, 95% CI: −0.45 to 0.31), and aerobic exercise vs. resistance training (SMD = −0.15, 95% CI: −0.57 to 0.27) all lacked statistical significance. These findings indicate that, although a gradient exists in the point estimates of effect sizes (aerobic exercise > mixed-modal training > HIIT > resistance training), based on the available evidence, no single exercise modality can be conclusively identified as having a clear superiority in improving working memory.

#### 3.3.5. Forest Plot Results

**Figure 4:**
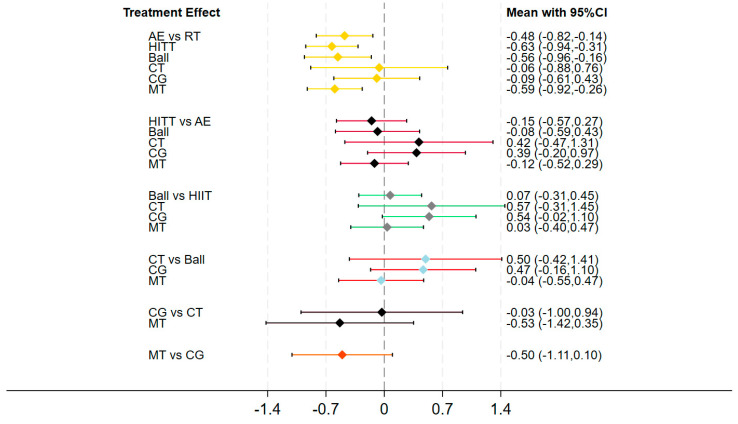
Forest plot of pairwise comparisons for working memory.

The forest plot (Figure 4) graphically summarizes effect estimates for each active intervention relative to control. The pattern is fully consistent with Section 3.3.3 and Table 2, where aerobic exercise, mixed-modal training, HIIT, and resistance training all showed significant benefits, while ball sports and cognitive training did not differ reliably from the control. Confidence intervals for ball sports and cognitive training were notably wider, reflecting limited studies and greater uncertainty. Pairwise comparisons among the four effective interventions revealed no statistically significant differences, underscoring that the apparent ranking hierarchy should be interpreted with caution.

#### 3.3.6. Publication Bias Assessment

**Figure 5:**
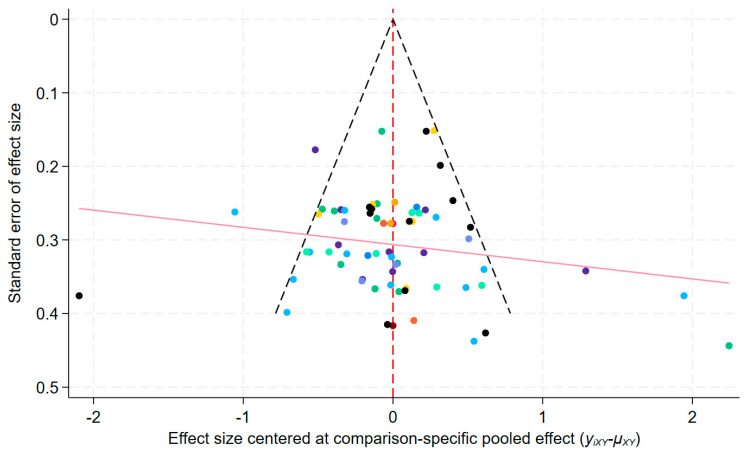
Comparison-adjusted funnel plot for assessing publication bias. Each color corresponds to studies from a specific pairwise comparison. Different colored dashed lines: These represent the comparison-specific pooled effect (centered at zero) for each pairwise comparison. The vertical line at x = 0: Indicates the null effect, where the study effect equals the comparison-specific summary effect.

Comparison-adjusted funnel plot inspection (Figure 5) revealed no obvious asymmetry indicative of publication bias. Most studies were concentrated in the middle and upper portions, and small-sample studies were roughly balanced. The slight positive slope in the regression line likely reflects clinical heterogeneity rather than publication bias.

#### 3.3.7. Contribution Analysis

The contribution analysis (Table 3) showed that control-group-referenced comparisons collectively contributed 58.9% of network evidence. A vs. G (aerobic vs. mixed modal) and DvsF (ball sports vs. control) had the highest individual contributions (13.9% each). DvsF had 100% self-contribution, anchoring the network despite only two studies. BvsG (resistance vs. mixed modal) had the lowest contribution (1.9%), reflecting reliance on indirect evidence. Direct evidence for comparisons such as BvsG, AvsE, and BvsE was limited; future RCTs should prioritize these understudied pairs.

The network exhibited two key characteristics: first, reference node dependence with higher precision in effect estimates for comparisons using the control group as the reference; and second, direct comparisons among interventions (AvsB, BvsC, BvsE, AvsE, AvsG, BvsG, etc.) collectively contributed 45.2%, indicating a more balanced information distribution compared to traditional star-shaped networks. Direct evidence for pairwise comparisons such as BvsG, AvsE, and BvsE was limited. Future research should prioritize conducting randomized controlled trials for these clinically relevant pairs with low direct evidence to optimize the network geometry.

### 3.4. Subgroup Analysis: Acute vs. Chronic Exercise Effects

To disentangle the temporal specificity of exercise-induced working memory enhancements, we performed separate network meta-analyses for acute (duration < 4 weeks) and chronic (duration ≥ 4 weeks) interventions. The detailed results are presented in Table 4 (acute) and Table 5 (chronic) (see Table 4 and Table 5).

#### 3.4.1. Acute Subgroup

The acute intervention network was fully connected and comprised six treatment nodes (aerobic, resistance, HIIT, cognitive, mixed, and passive control) with 19 studies contributing direct and indirect evidence. As shown in Table 4, resistance training (SMD = −0.49, 95% CI: −0.81 to −0.17, *p* = 0.003), HIIT (SMD = −0.40, 95% CI: −0.78 to −0.02, *p* = 0.038), and mixed training (SMD = −0.38, 95% CI: −0.70 to −0.07, *p* = 0.016) demonstrated statistically significant improvements over the passive control. Aerobic exercise approached significance (SMD = −0.35, *p* = 0.059), while cognitive training showed no reliable benefit (SMD = −0.07, *p* = 0.780). Resistance training had the highest probability of being the optimal intervention (P-score best = 39.9%). Ball sports were not represented in the acute subgroup.

#### 3.4.2. Chronic Subgroup

After excluding six single-arm studies to ensure network connectivity (see Section 2.5), the chronic intervention network was fully connected and included six nodes (aerobic, resistance, HIIT, ball sports, mixed, and passive control) with 10 studies. As shown in Table 5, resistance training (SMD = −0.58, 95% CI: −1.01 to −0.15, *p* = 0.008) and mixed training (SMD = −0.29, 95% CI: −0.56 to −0.02, *p* = 0.038) demonstrated statistically significant improvements relative to the passive control. HIIT exhibited the highest probability of being the optimal intervention (P-score best = 84.8%), although its effect versus control was not statistically significant (SMD = −0.06, *p* = 0.741). Aerobic exercise (SMD = −0.27, *p* = 0.178) and ball sports (SMD = 0.02, *p* = 0.915) did not differ significantly from control. The confidence intervals for several interventions in the chronic subgroup were notably wide (e.g., resistance training: 95% CI −1.01 to −0.15; ball sports: 95% CI −0.38 to 0.42), reflecting the reduced number of studies and the consequent imprecision of estimates. These findings should therefore be interpreted with appropriate caution.

#### 3.4.3. Comparative Summary

Comparing the two subgroups, three salient patterns emerge. First, resistance training consistently demonstrated significant benefits across both acute and chronic contexts, with moderate to large effect sizes (SMD = −0.49 and −0.58, respectively). Second, the probabilistic rankings differed, as resistance training ranked highest acutely, while HIIT ranked highest chronically despite its non-significant effect. Third, effect sizes in the chronic subgroup were generally larger than in the acute subgroup, suggesting that sustained training may confer more pronounced benefits.

It should be noted that the effect sizes in the subgroup analyses differ slightly in magnitude from those in the primary analysis. This is expected for two reasons. First, the subgroup analyses are based on subsets of studies with different compositions of intervention types and participant characteristics, which naturally yield different pooled estimates. Second, the exclusion of six single-arm studies from the chronic subgroup (see Section 2.5) further alters the evidence base. Importantly, the relative ordering of interventions and the pattern of statistical significance remain broadly consistent with the primary findings, supporting the robustness of the overall conclusions.

## 4. Discussion

This is the first network meta-analysis to systematically compare six exercise modalities on working memory in college students. Analysis of 35 RCTs (2314 participants) demonstrated that aerobic exercise, mixed-modal training, HIIT, and resistance training were significantly superior to the control (SMD = 0.48–0.63), whereas ball sports and cognitive training did not show significant effects. Subgroup analyses revealed that chronic interventions (≥4 weeks) produced substantially larger effect sizes than acute protocols, underscoring the importance of sustained training. Extending prior meta-analyses in older adults and children (Ludyga et al., 2020), aerobic-dominant modalities showed the highest probability of efficacy. However, direct comparisons among effective interventions revealed no statistically significant differences, suggesting that no single modality can be definitively identified as superior.

### 4.1. Mechanistic Considerations for Aerobic-Dominant Interventions

The findings support that working memory remains modifiable during early adulthood (Ferguson et al., 2021). Moderate effect sizes (SMD = 0.48–0.63) are lower than those reported in older adults (Northey et al., 2018), suggesting relatively lower neural adaptive reserve in college students. HIIT and mixed-modal training ranked highest in efficacy. Subgroup analyses further revealed that resistance training consistently demonstrated significant benefits across both acute and chronic contexts (SMD = −0.49 and −0.58, respectively), whereas HIIT—despite its top probabilistic ranking in the chronic subgroup (84.8%)—showed a small and non-significant effect (SMD = −0.06) in that context. This pattern echoes the ranking–significance discrepancy observed in the primary analysis and underscores the exploratory nature of probabilistic rankings. This pattern is compatible with a role for aerobic metabolic demand in promoting cognitive benefits. Potential mechanisms include increased expression of BDNF and VEGF, and enhanced cerebral hemodynamics (Stillman et al., 2020). Aerobic-dominant exercises promote sustained cerebrovascular stress and prefrontal perfusion (Joyner & Casey, 2015). However, because direct physiological measurements were not available in the included studies, any mechanistic interpretation remains speculative.

### 4.2. Interpreting the Discrepancy Between SUCRA Rankings and Pairwise Significance

A notable pattern requires careful interpretation. Resistance training demonstrated a statistically significant benefit versus control (SMD = −0.48, *p* = 0.006) yet ranked lowest among effective interventions (SUCRA = 13.7%). Conversely, ball sports showed no significant effect (*p* = 0.885) but ranked third (SUCRA = 70.1%). This apparent paradox stems from the fundamental difference between hypothesis testing and probabilistic ranking. SUCRA values integrate effect magnitude and precision (Rücker & Schwarzer, 2015). Interventions with large but imprecise estimates (e.g., ball sports, informed by only two studies) can achieve moderate rankings because the simulation assigns non-negligible probability to large-effect scenarios. In contrast, resistance training, though precisely estimated as significantly better than control, performed less favorably against other active interventions in the network. Critically, direct comparisons among effective interventions revealed no significant differences, indicating that the ranking hierarchy should not be interpreted as definitive evidence of superiority. SUCRA rankings are exploratory and hypothesis-generating, and decisions should be based on the full constellation of evidence.

### 4.3. The Limited Evidence for Ball Sports and Cognitive Training

Based on only two studies with wide confidence intervals, ball sports did not show a statistically significant effect, and no firm conclusions can be drawn regarding their efficacy. Several factors may contribute to this: tactical demands could compete for neural resources; included studies may have emphasized skill over aerobic load; and reaction time measures may not fully capture other executive function improvements. The null result for cognitive training (SMD = −0.09) was similarly inconclusive. While consistent with the hypothesis that pure cognitive tasks lacking metabolic stress are insufficient to induce gains (Melby-Lervåg & Hulme, 2013), the absence of a significant effect did not confirm this mechanism. Regarding broader theoretical implications, the present findings did not show a clear advantage for cognitively engaging activities over metabolically demanding ones. However, because direct comparisons among active interventions were not statistically significant, it would be premature to conclude that metabolic stress is more important than cognitive engagement. The pattern of effect sizes is compatible with a role for physiological mechanisms, but direct measurements are lacking; thus, any mechanistic interpretation remains speculative.

### 4.4. Mixed-Modal Training and HIIT: Balancing Efficiency and Accessibility

The high ranking of mixed-modal training (SUCRA = 76.0%) provides a flexible option for the reform of university physical education curricula. Its direct comparison with aerobic exercise showed no significant difference (SMD = −0.03), suggesting that as long as sufficient cardiopulmonary stimulation intensity is achieved, the specific organizational form may not be a determining factor. Modalities such as Tai Chi and circuit training align well with the Chinese cultural context and may produce synergistic effects that transcend single exercise types by simultaneously activating the aerobic metabolic system, neural efficiency processes related to motor learning, and cognitive-motor integration networks. Contribution analysis revealed that the aerobic–mixed comparison (AvsG) had the highest contribution (13.9%) with a self-contribution of 39.8%, indicating its core role in connecting these two key interventions. The moderate ranking of HIIT (60.3%) presents an interesting contrast to its time-efficiency advantage, as although single sessions of 20–30 min are more easily integrated into fragmented academic schedules, the intermittent load may be weaker than sustained steady-state stress in continuously activating neuroplasticity pathways. CvsF (HIIT vs. control) had a self-contribution of 44.5%, with more than half of its information (55.5%) originating from indirect pathways, reflecting the high connectivity of HIIT within the network—a characteristic that needs to be consolidated through more direct comparative studies in the future.

### 4.5. Interpretation of Heterogeneity and Inconsistency

The overall network heterogeneity was moderate (τ = 0.53, I^2^ ≈ 34%), suggesting that part of the between-study variance in effects originated from true variability rather than sampling error. The loop-specific inconsistency test revealed significant inconsistency in the A-B-G loop (resistance training–aerobic exercise–mixed-modal training; IF = 1.11, 95% CI: 0.15 to 2.08, *p* = 0.024). Possible reasons for this include: (1) this loop involved three active interventions, and its effect estimates may be substantially influenced by study characteristics (e.g., population baseline, intervention dosage); (2) the direct comparison between resistance training and aerobic exercise was based on only three studies, resulting in limited estimation precision; and (3) definitions of mixed-modal training may have varied across studies (e.g., Tai Chi vs. comprehensive physical fitness training). Given that neither the global inconsistency test (*p* = 0.535) nor the node-splitting analysis (all *p* > 0.05) detected significant inconsistency, and sensitivity analyses showed that removing studies related to this loop did not alter the core conclusions, the overall network estimates remain robust.

### 4.6. Methodological Considerations and Limitations

Several limitations warrant consideration. First, the network exhibited a star topology with sparse direct comparisons among active interventions; conclusions regarding exercise versus no exercise are relatively reliable, but determining the optimal type requires caution. Second, prioritizing reaction time may overlook strategic shifts reflected in accuracy and does not test whether resistance training selectively improves storage capacity. Third, although funnel plot inspection suggested no significant publication bias, the complexity of the network limits formal testing. The sample comprised healthy, highly educated students, limiting generalizability. Fourth, despite subgroup analysis by duration, residual heterogeneity within subgroups persists. Fifth, we could not definitively rule out speed–accuracy trade-offs, as accuracy data were not systematically extracted. Had accuracy data been available, a sensitivity analysis would have provided valuable insight; this remains an important direction for future work. Sixth, inconsistent reporting of frequency and intensity precluded formal meta-regression; future trials should adhere to standardized reporting guidelines to enable dose–response analyses. Seventh, the subgroup analyses, while conceptually justified, were based on reduced sample sizes (acute: *n* = 19 studies; chronic: *n* = 10 studies after excluding six single-arm trials to ensure network connectivity) and altered network geometries. In particular, the chronic subgroup included a different set of nodes (ball sports replacing cognitive training) and exhibited notably wide confidence intervals for several comparisons, reflecting the imprecision inherent in analyses with limited data. Consequently, the subgroup findings—especially the probabilistic rankings—should be interpreted as exploratory and hypothesis-generating, requiring confirmation in larger, dedicated trials.

### 4.7. Practical Implications and Future Directions

Preliminary evidence suggests that HIIT and mixed-modal training are promising time-efficient options, with traditional aerobic exercise as a foundational approach. Resistance training may be most beneficial when combined with cognitive tasks. These recommendations should be viewed as provisional given limited direct comparative evidence. Future research should: (1) conduct head-to-head RCTs for understudied comparisons; (2) employ neuroimaging to track neural mechanisms and distinguish acute versus chronic effects; (3) explore cognitive–motor dual-task training; (4) examine moderators such as baseline fitness; and (5) conduct long-term follow-up studies.

## 5. Conclusions

This network meta-analysis suggests that working memory in college students remains modifiable through structured exercise. First, interventions with substantial aerobic metabolic demand—HIIT, mixed-modal training, and aerobic exercise—were associated with moderate improvements in processing efficiency, with chronic protocols showing larger effects than acute exposures. Second, cognitively engaging activities alone (ball sports, cognitive training) did not confer reliable benefits, though limited data preclude firm conclusions. Third, while probabilistic rankings favored HIIT and mixed-modal training, direct comparisons among effective interventions revealed no statistically significant differences; thus, the optimal exercise type cannot be definitively established. For practice, aerobic-dominant programs are recommended as a foundational strategy, with resistance training best combined with cognitive tasks. Future research should prioritize head-to-head comparisons and incorporate both reaction time and accuracy metrics to clarify the nature of exercise-induced cognitive changes.

## Figures and Tables

**Table 1:** Network meta-analysis ranking results of different exercise interventions on working memory.

Intervention Type	Compared with Control Group SMD (95%CI)	*p*	SUCRA (%)	Probability of Being Best (%)	Mean Rank
HIIT	−0.56 (−0.96~−0.16)	0.007	80.3	32.5	2.2
Mixedmixed-modal training	−0.59 (−0.92~−0.26)	<0.001	76.0	29.5	2.4
Ball sports	−0.06 (−0.88~0.76)	0.885	70.1	22.1	2.8
Aerobic exercise	−0.63 (−0.94~−0.32)	<0.001	60.3	8.5	3.4
Cognitive training	−0.09 (−0.61~0.43)	0.733	26.2	6.5	5.4
Control group	—	—	23.4	0.9	5.6
Resistance training	−0.48 (−0.82~−0.14)	0.006	13.7	0.0	6.2

Note: SUCRA, surface under the cumulative ranking curve; based on 5000 Monte Carlo simulation samples. SMD, standardized mean difference; negative values indicate that the intervention is superior to the control group (i.e., reduced reaction time).

**Table 2:** League table of pairwise comparisons among all interventions; standardized mean differences (SMD) with 95% confidence intervals.

**Aerobic** **exercise**						
**−0.03 (−0.47, 0.40)**	**Mixed-modal** **training**					
**−0.07 (−0.45, 0.31)**	**−0.04 (−0.55, 0.47)**	**HIIT**				
**−0.15 (−0.57, 0.27)**	**−0.12 (−0.52, 0.29)**	**−0.08 (−0.58, 0.42)**	**Resistance** **training**			
**−0.57 (−1.45, 0.31)**	**−0.53 (−1.42, 0.35)**	**−0.50 (−1.41, 0.42)**	**−0.42 (−1.30, 0.47)**	**Ball sports**		
**−0.54 (−1.10, 0.02)**	**−0.50 (−1.11, 0.10)**	**−0.47 (−1.10, 0.16)**	**−0.39 (−0.97, 0.20)**	**0.03 (−0.94, 1.00)**	**Cognitive** **training**	
**−0.63 (−0.94, −0.31)**	**−0.59 (−0.92, −0.26)**	**−0.56 (−0.96, −0.16)**	**−0.48 (−0.82, −0.14)**	**−0.06 (−0.88, 0.76)**	**−0.09 (−0.60, 0.43)**	**Control group**

**Table 3:** Contribution analysis of direct comparisons in the network.

Direct Comparison	Number of Studies	Network Contribution (%) a	Self-Contribution (%) b	Key Characteristics
A vs. G	5	13.9	39.8	Highest contribution; connects aerobic and mixed-modal training
D vs. F	2	13.9	100.0	Basic contrast; anchors network structure
C vs. F	7	12.0	44.5	High indirect dependence (55.5%)
F vs. G	12	10.7	34.8	Control group vs. mixed-modal training
A vs. B	3	9.3	29.3	Local precision for resistance–aerobic comparison
B vs. C	9	9.2	31.6	Local precision for resistance–HIIT comparison
E vs. F	4	8.8	30.6	Cognitive training vs. control group
A vs. F	10	8.1	23.8	Resistance training vs. control group
B vs. E	2	6.4	22.9	Limited evidence
B vs. F	13	5.4	16.2	Resistance training vs. control group
A vs. E	1	4.5	23.8	Limited evidence
B vs. G	1	1.9	5.1	Lowest contribution; single-arm comparison

Note: a = Network contribution represents the percentage contribution of each direct comparison to the entire network estimate. b = Self-contribution represents the percentage contribution of each direct comparison to its own mixed estimate (i.e., diagonal values from the percentage contribution matrix). A = aerobic training, B = resistance training, C = HIIT, D = ball sports, E = cognitive training, F = control group, G = mixed-modal training.

**Table 4:** Network meta-analysis results for the acute subgroup.

Intervention	SMD (vs. Control)	95% Confidence Interval	*p*	P-Score (Best Rank %)
Aerobic Exercise	−0.35	(−0.72, 0.01)	0.059	14.1
Resistance Training	−0.49	(−0.81, −0.17)	0.003	39.9
HIIT	−0.40	(−0.78, −0.02)	0.038	22.7
Cognitive Training	−0.07	(−0.57, 0.43)	0.780	2.9
Mixed Training	−0.38	(−0.70, −0.07)	0.016	20.3

Note: Negative SMD values indicate shorter reaction time (better performance) relative to passive control.

**Table 5:** Network meta-analysis results for the chronic subgroup.

Intervention	SMD (vs. Control)	95% Confidence Interval	*p*	P-Score (Best Rank %)
Aerobic Exercise	−0.27	(−0.65, 0.12)	0.178	—
Resistance Training	−0.58	(−1.01, −0.15)	0.008	0.7
HIIT	−0.06	(−0.43, 0.30)	0.741	84.8
Ball Sports	0.02	(−0.38, 0.42)	0.915	2.4
Mixed Training	−0.29	(−0.56, −0.02)	0.038	10.7

Note: Negative SMD values indicate shorter reaction time (better performance) relative to passive control.

## Data Availability

The original contributions presented in this study are included in the article. Further inquiries can be directed to the corresponding author.

## Appendix A

1. Audiffren, M., Tomporowski, P. D., & Zagrodnik, J. (2009). Acute aerobic exercise and information processing: Modulation of executive control in a Random Number Generation task. *Acta Psychologica*, *132*(1), 85–95. https://doi.org/10.1016/j.actpsy.2009.06.008.
2. Cai, Z. D., Shu, Z., Wang, X., & Jiang, W. T. (2023). Jixing danlida yundong dui gaoling laonianren gongzuojiyi de yingxiang: Laizi fNIRS de zhengju [Effects of acute elastic band exercise on working memory in the oldest-old: Evidence from fNIRS]. *Zhongguo Tiyu Keji [China Sport Science and Technology]*, *59*(4), 45–52.
3. Chen, A. G., Yin, H. C., Yan, J., & Yang, Y. (2011). Butong qiangdu duanshi youyang yundong dui zhixing gongneng de yingxiang [Effects of acute aerobic exercise of different intensities on executive function]. *Xinli Xuebao [Acta Psychologica Sinica]*, *43*(9), 1055–1062.
4. De Rosa, O., D’Onofrio, P., Conte, F., De Luca, P., Schiavone, C., Malloggi, S., Giganti, F., & Ficca, G. (2024). Video gaming impact on adults’ psychological well-being cognitive functions and sleep. *Journal of Sleep Research*, *33*, e14291. https://doi.org/10.1111/jsr.14291.
5. Ekerer, C., Ince, G., & Over, M. F. (2025). The effect of structured brain gym and brisk walking training on the executive functions of university students: A single-blinded randomised controlled trial. *International Journal of Sport and Exercise Psychology*, *23*(5), 724–741. https://doi.org/10.1080/1612197X.2024.2357277.
6. Erickson, K. I., Voss, M. W., Prakash, R. S., Basak, C., Szabo, A., Chaddock, L., Kim, J. S., Heo, S., Alves, H., White, S. M., Wojcicki, T. R., Mailey, E., Vieira, V. J., Martin, S. A., Pence, B. D., Woods, J. A., McAuley, E., & Kramer, A. F. (2011). Exercise training increases size of hippocampus and improves memory. *Proceedings of the National Academy of Sciences of the United States of America*, *108*(7), 3017–3022. https://doi.org/10.1073/pnas.1015950108.
7. Heitz, R. P. (2014). The speed-accuracy tradeoff: History, physiology, methodology, and behavior. *Frontiers in Neuroscience*, *8*, 150. https://doi.org/10.3389/fnins.2014.00150.
8. Li, L., Cui, J., Xiang, Q., & Fu, H. (2020). 8 zhou butong leixing de yundong dui nüdaxuesheng zhixing gongneng de yingxiang [Effects of 8 weeks of different types of exercise on executive function in female college students]. *Zhongguo Yundong Yixue Zazhi [Chinese Journal of Sports Medicine]*, *39*(10), 810–816. https://doi.org/10.16038/j.1000-6710.2020.10.010.
9. Li, L., Yuan, J. J., Ji, T., Zhou, Z. H., Cui, J., & Ji, L. (2014). Duanshi zhongdeng qiangdu youyang yundong dui nüdaxuesheng zhuanhuan gongneng de fMRI yanjiu [An fMRI study on the effect of short-term moderate-intensity aerobic exercise on shifting function in female college students]. *Beijing Tiyu Daxue Xuebao [Journal of Beijing Sport University]*, *37*(12), 56–60. https://doi.org/10.19582/j.cnki.11-3785/g8.2014.12.010.
10. Liu, J. Y. (2017). Youyang tiyu duanlian yu daxuesheng zhixing gongneng guanxi de “jiliang xiaoying” [The “dose-response” relationship between aerobic exercise and executive function in college students]. *Beijing Tiyu Daxue Xuebao [Journal of Beijing Sport University]*, *40*(1), 58–64. https://doi.org/10.19582/j.cnki.11-3785/g8.2017.01.010.
11. Ludyga, S., Gerber, M., Brand, S., Pühse, U., & Colledge, F. (2018). Effects of aerobic exercise on cognitive performance among young adults in a higher education setting. *Research Quarterly for Exercise and Sport*, *89*(2), 164–172. https://doi.org/10.1080/02701367.2018.1438575.
12. Lupien, S. J., McEwen, B. S., Gunnar, M. R., & Heim, C. (2009). Effects of stress throughout the lifespan on the brain, behaviour and cognition. *Nature Reviews Neuroscience*, *10*(6), 434–445. https://doi.org/10.1038/nrn2639.
13. Ma, Q., Hou, H. S., & Li, J. X. (2020). Gaohai huanjing zhong duanshi youyang yundong yu daxuesheng zhixing gongneng de shiyan yanjiu [An experimental study on short-term aerobic exercise and executive function of college students in high-altitude environment]. *Zhongyang Minzu Daxue Xuebao (Ziran Kexue Ban) [Journal of Minzu University of China (Natural Sciences Edition)]*, *29*(1), 81–86.
14. Moreau, D. (2015). Brains and brawn: Complex motor activities to maximize cognitive enhancement. *Educational Psychology Review*, *27*(3), 475–482. https://doi.org/10.1007/s10648-015-9323-5.
15. Mou, H., Fang, Q., Tian, S. D., & Qiu, F. H. (2023). Effects of acute exercise with different modalities on working memory in men with high and low aerobic fitness. *Physiology & Behavior*, *258*, 114012. https://doi.org/10.1016/j.physbeh.2022.114012.
16. Peng, Y. F. (2015). Jixing youyang yundong dui nüdaxuesheng shuaxin gongneng de yingxiang yanjiu [Effect of acute aerobic exercise on updating function of female college students]. *Dangdai Tiyu Keji [Contemporary Sports Technology]*, *5*(27), 30–31. https://doi.org/10.16655/j.cnki.2095-2813.2015.27.030.
17. Peng, Y. F., & Zhou, C. L. (2016). Jixing youyang yundong dui nüdaxuesheng gongzuojiyi yingxiang de shicheng tedian [The time-course characteristics of the effect of acute aerobic exercise on working memory in female college students]. *Zhongguo Yundong Yixue Zazhi [Chinese Journal of Sports Medicine]*, *35*(5), 473–477. https://doi.org/10.16038/j.1000-6710.2016.05.011.
18. Qi, L. Q., Cui, L., Zhang, J. Y., Wang, D. L., Luo, R., Xin, Z. L., & Yin, H. C. (2022). Taiji (bafa wubu) dui daxuesheng shuaxin gongneng de yingxiang: Laizi zinao fashenjing huodong de zhengju [Effects of Tai Chi (Ba Fa Wu Bu) on updating function in college students: Evidence from spontaneous brain neural activity]. *Tiyu Kexue [China Sport Science]*, *42*(10), 35–45. https://doi.org/10.16469/j.css.202210005.
19. Rehfeld, K., Müller, P., Aye, N., Schmicker, M., Dordevic, M., Kaufmann, J., Hökelmann, A., & Müller, N. G. (2017). Dancing or fitness sport? The effects of two training programs on hippocampal plasticity and balance abilities in healthy seniors. *Frontiers in Human Neuroscience*, *11*, 305. https://doi.org/10.3389/fnhum.2017.00305.
20. Shields, N., Mizzi, N., Buhlert-Smith, K., Strydom, A., Prendergast, L., & Hocking, D. R. (2022). A 12-week exercise programme has a positive effect on everyday executive function in young people with Down syndrome: A pilot non-randomised controlled trial. *Journal of Intellectual Disability Research*, *66*(12), 924–938. https://doi.org/10.1111/jir.12979.
21. Si, H. Q., Yan, C., Zhu, T., Liu, M. Q., Yang, Z. Y., Hu, N. Y., Qin, Y., & Mu, L. (2025). Effects of variable-intensity interval training and moderate-intensity continuous aerobic exercise on executive function and underlying neural mechanisms in male college students. *Frontiers in Psychology*, *16*, 1643698. https://doi.org/10.3389/fpsyg.2025.1643698.
22. Su, R., Zhu, T., Peng, B., Tao, W. J., & Wang, D. S. (2021). Yinyue lianhe youyang yundong dui daxuesheng zhixing gongneng de cujin xiaoyi [The promoting effect of music combined with aerobic exercise on executive function of college students]. *Zhongguo Tiyu Keji [China Sport Science and Technology]*, *57*(8), 88–95. https://doi.org/10.16470/j.csst.2020060.
23. Wang, L., Shi, L. Y., Tian, Y., Yu, H., & Qi, H. X. (2020). Zhongdeng qiangdu youyang tiyu duanlian dui daxuesheng gongzuojiyi yingxiang de yanjiu [Effect of moderate-intensity aerobic exercise on working memory of college students]. *Dangdai Tiyu Keji [Contemporary Sports Technology]*, *10*(27), 25–29. https://doi.org/10.16655/j.cnki.2095-2813.2004-5797-7593.
24. Wang, Y., Ji, Q., Zhou, C., & Wang, Y. (2022). Brain mechanisms linking language processing and open motor skill training. *Frontiers in Human Neuroscience*, *16*, 911894. https://doi.org/10.3389/fnhum.2022.911894.
25. Wang, Y., Tian, J., & Yang, Q. X. (2023). Tai Chi exercise improves working memory capacity and emotion regulation ability. *Frontiers in Psychology*, *14*, 1047544. https://doi.org/10.3389/fpsyg.2023.1047544.
26. Wilckens, K. A., Woo, S. G., Kirk, A. R., Erickson, K. I., & Wheeler, M. E. (2014). Role of sleep continuity and total sleep time in executive function across the adult lifespan. *Psychology and Aging*, *29*(3), 658–665. https://doi.org/10.1037/a0037234.
27. Yang, R. Z., & He, Z. H. (2023). HIIT he MICT youyong yundong dui daxuesheng zhixing gongneng de yingxiang [Effects of HIIT and MICT swimming on executive function in college students]. *Keji Zixun [Science & Technology Information]*, *21*(2), 240–243. https://doi.org/10.16661/j.cnki.1672-3791.2204-5042-2375.
28. Yang, Y. T., Luo, J. J., & Huang, H. (2025). Tiyu duanlian dui daxuesheng zhixing gongneng de yingxiang: Lvse huanjing he duanlian shichang de zuoyong [The effect of physical exercise on executive function in college students: The role of green environment and exercise duration]. *Xiandai Yufang Yixue [Modern Preventive Medicine]*, *52*(14), 2625–2630. https://doi.org/10.20043/j.cnki.MPM.202501326.
29. Yang, Y. T., Wan, M., & Wan, X. Q. (2021). Daqiangdu jianxie yundong yu zhongdeng qiangdu chixu youyang yundong dui daxuesheng zhixing gongneng de yingxiang [Effects of high-intensity interval training and moderate-intensity continuous aerobic exercise on executive function in college students]. *Tianjin Tiyu Xueyuan Xuebao [Journal of Tianjin University of Sport]*, *36*(6), 733–738. https://doi.org/10.13297/j.cnki.issn1005-0000.2021.06.017.
30. Yu, Q. (2023). Duanshi youyang yundong dui chaozhong daxuesheng zhixing gongneng xiaoyi de yingxiang [Effect of short-term aerobic exercise on executive function benefits in overweight college students]. *Jiamusi Zhiye Xueyuan Xuebao [Journal of Jiamusi Vocational Institute]*, *39*(11), 82–84.
31. Yuan, S. J., Lu, C. F., Ma, Y., & Luo, X. B. (2024). “Dupin” haishi “liangyao”? Dianzi jingji dui daxuesheng zhixing gongneng yingxiang de shijian jiliang xiaoying [“Drug” or “medicine”? Time dose effect of e-sports on college students’ executive function]. *Wuhan Tiyu Xueyuan Xuebao [Journal of Wuhan Sports University]*, *58*(8), 73–80. https://doi.org/10.15930/j.cnki.wtxb.2024.08.007.
32. Yue, T., Liu, L., Nitsche, M. A., Kong, Z. W., Zhang, M., & Qi, F. X. (2024). Effects of high-intensity interval training combined with dual-site transcranial direct current stimulation on inhibitory control and working memory in healthy adults. *Human Movement Science*, *96*, 103240. https://doi.org/10.1016/j.humov.2024.103240.
33. Zhao, Q., Ding, H., Wang, P., Li, S. F., Jia, S. Q., Liu, Q., & Chen, Q. J. (2025a). 8 zhou zhongqiangdu pingpangqiu yundong dui yiyu zhengzhuang daxuesheng yanyu gongzuojiyi de yingxiang—Laizi ERP de zhengju [Effect of 8-week moderate-intensity table tennis exercise on verbal working memory in college students with depressive symptoms: Evidence from ERPs]. *Shanghai Tiyu Daxue Xuebao [Journal of Shanghai University of Sport]*, *49*(8), 88–98. https://doi.org/10.16099/j.sus.2025.01.02.0002.
34. Zhao, Q., Wang, P., Jia, S. Q., Liu, Q., Liu, C., Li, S. F., Liu, W. Z., & Mao, L. J. (2025b). Danci butong qiangdu pingpangqiu yundong dui yiyu zhengzhuang daxuesheng gongzuojiyi ji shijian guanlian dianwei de yingxiang [Effect of a single session of table tennis exercise at different intensities on working memory and event-related potentials in college students with depressive symptoms]. *Haijun Junyi Daxue Xuebao [Academic Journal of Naval Medical University]*, *46*(7), 898–909. https://doi.org/10.16781/j.CN31-2187/R.20250214.
35. Zhu, L. N., Yin, H. C., Chen, A. G., Zhang, H. D., Xiong, X., Chen, D. D., Cui, L., & Wang, Y. (2020). Daxuesheng youyang yundong nengli yu zhixing gongneng de guanxi: Piceng houdu de zhongjie zuoyong [Relationship between aerobic fitness and executive function in college students: The mediating role of cortical thickness]. *Zhongguo Tiyu Keji [China Sport Science and Technology]*, *56*(11), 48–54. https://doi.org/10.16470/j.csst.2020127.
